# Rare single‐nucleotide variants of *MLH1* and *MSH2* genes in patients with Lynch syndrome

**DOI:** 10.1002/cnr2.1930

**Published:** 2023-11-02

**Authors:** Seyed Mohsen Mirabdolhosseini, Mohammad Yaghoob Taleghani, Leili Rejali, Hossein Sadeghi, Nayeralsadat Fatemi, Mehdi Tavallaei, Amin Famil Meyari, Narges Saeidi, Pardis Ketabi Moghadam, Amir Sadeghi, Hamid Asadzadeh Aghdaei, Mohammad Reza Zali, Ehsan Nazemalhosseini Mojarad

**Affiliations:** ^1^ Basic and Molecular Epidemiology of Gastrointestinal Disorders Research Center Research Institute for Gastroenterology and Liver Diseases, Shahid Beheshti University of Medical Sciences Tehran Iran; ^2^ Genomic Research Center Shahid Beheshti University of Medical Sciences Tehran Iran; ^3^ Department of Colorectal Surgery Medical Science of Shahid Beheshti University Tehran Iran; ^4^ Gastroenterology and Liver Diseases Research Center Research Institute for Gastroenterology and Liver Diseases, Shahid Beheshti University of Medical Sciences Tehran Iran

**Keywords:** non‐pathogenic variants, Lynch syndrome, *MLH1*, *MSH2*, pathogenic variants

## Abstract

**Background:**

Approximately 5% of colorectal cancers (CRCs) are hereditary. Lynch syndrome (LS), also known as hereditary nonpolyposis colorectal cancer (HNPCC), is the most common form of recognized hereditary CRC. Although Iran, as a developing country, has a high incidence of CRC, the spectrum of variants has yet to be thoroughly investigated.

**Aims:**

This study aimed to investigate pathogenic and non‐pathogenic variants in *MLH1* and *MSH2* genes in Iranian patients with suspected Lynch syndrome (sLS).

**Methods and results:**

In the present study, 25 peripheral blood samples were collected from patients with sLS and high microsatellite instability (MSI‐H). After DNA extraction, all samples underwent polymerase chain reaction and Sanger sequencing to identify the variants in the exons of *MLH1* and *MSH2* genes. The identified variants were interpreted using prediction tools, and were finally reported under ACMG guidelines. In our study population, 13 variants were found in the *MLH1* gene and 8 in the *MSH2* gene. Interestingly, 7 of the 13 *MLH1* variants and 3 of the 8 *MSH2* variants were novel, whereas the remaining variants were previously reported or available in databases. In addition, some patients with sLS did not have variants in the exons of the *MLH1* and *MSH2* genes. The variants detected in the *MLH1* and *MSH2* genes had specific characteristics regarding the number, area of occurrence, and their relationship with demographic and clinicopathologic features.

**Conclusion:**

Overall, our results suggest that analysis of *MLH1* and *MSH2* genes alone is insufficient in the Iranian population, and more comprehensive tests are recommended for detecting LS.

## 1 INTRODUCTION

Colorectal cancer (CRC) is a major global health issue, particularly in developing countries.^1^, ^2^, ^3^ This type of cancer is divided into three subclasses: sporadic, familial, and hereditary.^4^ The sporadic form of CRC, which affects 80% of CRC patients, is primarily caused by somatic changes in nucleotide and protein levels, as well as lifestyle and epigenetic factors. The second most common type (15%–20%) is familial CRC, in which at least one blood relative has CRC. Finally, the least common form is hereditary CRC, which is caused by inherited germline variants in cancer predisposition genes.^5^, ^6^, ^7^

Lynch syndrome (LS), also known as hereditary non‐polyposis colorectal cancer (HNPCC), is a typical example of a hereditary CRC. In addition to LS, there are other hereditary CRC syndromes, such as familial adenomatous polyposis (FAP) and MUTYH‐associated polyposis (MAP). However, LS is the most common type of hereditary CRC and accounts for 3%–5% of all CRC cases.^8^ LS is inherited in an autosomal dominant manner and is associated with a high risk of early onset cancers such as CRC, neoplastic lesions, and microsatellite instability (MSI). The occurrence of LS is known to be linked to germline variants in mismatch repair (MMR) genes, including MLH1 (MIM# 120436), MSH2 (MIM# 120435), PMS2 (MIM# 600259), MSH6 (MIM# 600678). In addition to the four mentioned genes, deletion of EPCAM (MIM# 185535), can have a disease‐causing effect by causing structural abnormalities on chromosome position 2p21 due to its proximity to the *MSH2* gene.^9^, ^10^

It is currently recognized that the MMR system plays an essential role in maintaining genomic stability by removing replication errors from DNA. In this system, each MMR protein has a unique functional domain and, of course, a specific function. Monoallelic variants in each of the MMR genes may cause LS as a consequence of MMR deficiency. Pathogenic variants in DNA sequences related to the functional domain of MMR genes may impair their normal repair ability, which will result in MSI and tumorigenesis.^11^, ^12^ Regarding the identified variants, it is assumed that these alterations follow particular patterns. For example, in LS, approximately 60%–70% of the detected variants occur in the *MLH1* and *MSH2* genes.^13^ It is already well known that the types of variants in *MLH1* and *MSH2* genes may vary in different races and regions of the world and even within countries themselves, therefore this challenge requires further investigation.^14^

Iran, as a developing nation, has witnessed an increase in the incidence and mortality rates of CRC over the past few decades.^15^, ^16^, ^17^ Although we have reliable information about the incidence of CRC in the Iranian population, there is a lack of information regarding the proportion of inherited routes that drive people to CRC or other Lynch‐related syndromes.^18^ This data shortage is relevant to the different angles of LS, such as incidence, type of variants, hotspot exons, typical alterations, and pathogenic or non‐pathogenic variations in the Iranian population. Regarding the investigations that have been done about LS in the Iranian population, valuable results have been achieved. Recently, a new suspected disease‐causing abnormality in the MAP3K1 gene has been reported by Fatemi et al.^19^ To summarize, there are still conflictions and unknown aspects about LS in the Iranian population, so to fill this gap and be able to clearly compare the variant's characteristics of the Iranian population and the likely effects with those of other races and populations, we sought to evaluate individuals with sLS from a variety of perspectives in this project, including demographic, bioinformatics, molecular, and pathological features, to have a comprehensive look at this syndrome in the sLS cases.

## 2 MATERIALS AND METHODS

### 2.1 Samples and DNA extraction

Inclusion criteria, like Amsterdam and Bethesda, which both depend on clinical data and family history, are used to categorize people with sLS. The Bethesda guidelines also consider the MSI tumor marker, a sensitive and cost‐effective strategy for tumor testing.^20^ Therefore, the revised Bethesda guideline was used as the selected criteria. Twenty‐five patients, who were referred to the Hospital due to sLS with MSI‐H, were subjected to the monitoring of variants in the *MLH1* and *MSH2* genes. Written informed consent was obtained for participation in this study. Documents of the included patients were available, and clinicopathological, demographic, and MSI status results were extracted (MSI testing had been done previously for these patients and the results were available in their documents (The markers were included: BAT25, BAT26, NR21, NR24, and NR27). After studying the patient's records and passing the revised Bethesda necessities, total genomic DNA was extracted from peripheral blood lymphocytes using a Blood DNA genomic extraction mini kit (QIAamp DNA Mini Kit) according to the manufacturers' directions.

### 2.2 *MLH1* and *MSH2* screening

All exons of the *MLH1* and *MSH2* genes were amplified by polymerase chain reaction (PCR). Primer pairs were designed with “Gene runner” software. Primer sequences are presented in Table 1. 50–100 ng of genomic DNA was amplified in a 25 μL reaction volume using 1.5 mM MgCl2, 0.2 mM dNTP, 0.2 μM of each primer, and 1.25 units of Taq DNA polymerase. Cycling conditions were as follows: 95°C for 5 min, 95°C for 1 min/1 min for annealing/72°C for 1 min (×33 cycles), followed by 10 min at 72°C. The annealing temperature was different in each PCR amplification depending on the primer sequence. The PCR products were subjected to automated cycle sequencing using the Big Dye terminator cycle sequencing kit v.3.1 (Applied Biosystems, Foster City, CA, USA) and capillary gel electrophoresis on the ABI 3130xl genetic analyzer (Applied Biosystems).

**TABLE 1:** List of primer sequences used for *MLH1* and *MSH2* analysis in this study.

*MSH2*			*MLH1*		
Exon no.	Direction	Sequences	Exon no.	Direction	Sequences
EXON1	Forward	CGTCTGCTTATGATTGGTTG	Exon1	Forward	AGGCACTTCCGTTGAGCATC
	Reverse	GACGTAAACACTCCGTGATC		Reverse	CACACGGTCTGCGGAAAAG
EXON2	Forward	ATCAGCAGCATGAAGTCCAG	Exon2	Forward	TGGAGTTTGTTATCATTGCTTGG
	Reverse	CCATTCTACTATCACAATCTAC		Reverse	ATACCCATTTTGTCTCCCACC
EXON3	Forward	TAATAAGGTTCATAGAGTTTGG	Exon3	Forward	AGATCTCGCCACTGCACTTC
	Reverse	AGTATCATGTCAATTAAAGAGC		Reverse	TCATAGGTGGTACTGGGTACTTCC
EXON4	Forward	AAAGAGTTGTTACCGTTGGGAC	Exon4	Forward	TTCAGATAACCTTTCCCTTTGG
	Reverse	TTGATACACAGTTTAGGTTTTGAG		Reverse	AGCAATACCCCAACTGAAGG
EXON5	Forward	GTTTGGATTGGGAAGGAACAC	Exon5	Forward	GGAAGTAGTGGAGAAATAAACAGG
	Reverse	GCTTCTTCAGTATATGTCAATG		Reverse	ATGCCACAAAAGCCAATAGTC
EXON6	Forward	GTTTTTCATGGCGTAGTAAGG	Exon6	Forward	GCTTTTGCCAGGACATCTTG
	Reverse	ACTCTATTACTATGTACTCTG		Reverse	TGGGGAGATGAGAGAAACTGC
EXON7	Forward	GCTGATTTAGTTGAGACTTAC	Exon7,8	Forward	AACTAAAAGGGGGCTCTGAC
	Reverse	AGTCACCACCACCAACTTTATG		Reverse	AAAGGTTCCAAAATAATGTGATG
EXON8	Forward	TTTGAGTGCTACATCATCTCC	Exon9	Forward	AATGGATGGATGAATGGACAGG
	Reverse	ATCCACTGTCCACAAAGGTG		Reverse	GTGGGTGTTTCCTGTGAGTGG
EXON9	Forward	ACATCATCAGCACTGTAACTG	Exon10	Forward	GTGGCGACAGGTAAAGGTGC
	Reverse	ATGTGAAGTCATCATCTTGGG		Reverse	GACAGAACATCCTTTTGCCAGTG
EXON10	Forward	AACATTCATAAGGGAGTTAAGG	Exon11	Forward	TCATCTGGCCTCAAATCTTCTGG
	Reverse	TCAATGGAGAACAGACGGAC		Reverse	AAATCTGGGCTCTCACGTCTGG
EXON11	Forward	TGTCCCTAAGGAGTTGTTCG	Exon12	Forward	CATTTGGGGACCTGTATATC
	Reverse	AGTCAGAATGTAATGGCTTGC		Reverse	AGTTAGAAGGCAGTTTTATTACAG
EXON12	Forward	TATGTTGAGTTTTAGGTGGGTTC	Exon13	Forward	CTCCTCCAAAATGCAACCCAC
	Reverse	CCTTCTAAATCTTCCCTCTAAAC		Reverse	TCTGACAACATGACTGCTTTCTCC
EXON13	Forward	TCATCAGTGTACAGTTTAGGAC	Exon14	Forward	GTTCTGGTGCCTGGTGCTTTGG
	Reverse	ACAAAGTATATAAAGTCCACAGG		Reverse	GCTTTTGTGCCTGTGCTCCCTG
EXON14	Forward	GCATATCCTTCCCAATGTATTG	Exon15	Forward	ATGTTTCAGGGATTACTTCTC
	Reverse	ATGCTTTAGAATGAGTGGTCC		Reverse	ACATTTGTTTAAGTTGGCTACC
EXON15	Forward	TACAGCACTGTGTGCCAAGT	Exon16	Forward	CATTCTGATAGTGGATTCTTGG
	Reverse	AACCTTCATCTTAGTGTCCTG		Reverse	CAAGTTATCTGCCCACCTC
EXON16	Forward	ATGTGTGATATGTTTAGATGG	Exon17,18	Forward	GGGAAAGCACTGGAGAAATGG
	Reverse	AGTCCTCAGTTACAGCTCTC		Reverse	TCCTGTCCTAGTCCTGGGGTG
			Exon19	Forward	AAAAAATCCTCTTGTGTTCAG
				Reverse	GGAATACAGAGAAAGAAGAACAC

### 2.3 In silico analysis

The following online software was used to determine the pathogenicity of each variant: SIFT (https://sift.bii.a-star.edu.sg/), CADD (https://cadd.gs.washington.edu/), ClinVar (https://www.ncbi.nlm.nih.gov/clinvar), PolyPhen (http://genetics.bwh.harvard.edu/pph/), PROVEAN (http://provean.jcvi.org/genome_submit_2.php?species=human), REVEL (https://www.ensembl.org/index.html), MetaLR (https://www.ensembl.info/tag/metalr/), and Mutational Assessor (http://mutationassessor.org/r3/).

Finally, the results were analyzed for demographic and clinicopathologic features, and their pathogenicity was evaluated by the American College of Medical Genetics and Genomics (ACMG) guidelines and gathered in Table 2.^21^ The outcomes were compared with other available results in different regions, races, public databases, and evaluations of software analysis.

**TABLE 2:** Comparison of clinicopathological and demographic data with variants status of patients with sLS.

Comparison of clinicopathological and demographic data													
Patients ID	Af/3	Ay/6	Ba/8	Em/10	Fk/11	he/14	Hh/15	Lm/16	Lr/17	Ma/19	Mh/20	Mm/21	mh/23
Sex	M	F	F	M	M	M	M	F	M	F	F	F	M
Tumor location	Ascending colon‐Right‐	Rectusigmoid‐Left‐	Rectum‐Left‐	Rectusigmoid‐Left‐	Rectum‐Left‐	Rectum‐Left‐	Ascending colon‐Right‐	Rectum‐Left‐	Splenic flexure‐Left‐	Ascending colon‐Right‐	Secum‐Right‐	Secum‐Right‐	Rectum‐Left‐
Differentiation	Well	Moderate	Well	Well	Well	Moderate	Well	Well	Well	Moderate	Well	Poor	Poor
TNM	M1/N1/T2	M0/N0/T3	M0/N0/T3	M0/N0/T3	M0/N0/T3	M0/N0/T3	M0/N1/T3	n/a	M0/N0/T3	M0/N2/T3	M0/N1/T1	M1/N2/T3	M0/N2/T3
Stage	IV	IIA	IIA	IIA	IIA	IIA	IIIB	I	IIA	IIIB	IIIA	IV	IIIC
MSI status	MSI‐H	MSI‐H	MSI‐H	MSI‐H	MSI‐H	MSI‐H	MSI‐H	MSI‐H	MSI‐H	MSI‐H	MSI‐H	MSI‐H	MSI‐H
Diagnosis age	52	48	42	39	48	48	37	27	26	34	57	48	41
Live/death	D	D	L	L	L	L	L	L	L	L	L	L	L
*MLH1* variants	c.1612 T > G c.1592 T > G	c. 1612 T > c.1592 T > G	c.1612 T > G c.1592 T > G c.655A > G	c.655A > G c.1969A > T	c. 1612 T > G c.1592 T > G	c. 1612 T > G c.1592 T > G c.394G > A	c.1612 T > G c.1592 T > G	c.2002G > A c.2005G > A	c.1612 T > G c.1592 T > G c.2002G > A c.2005G > A	c.2002G > A c.2005G > A c.655A > G	c.1612 T > G c.1592 T > G c.2002G > A c.2005G > A	c.655A > G	c.1612 T > G c.1592 T > G c.2002G > A c.2005G > A c.977 T > C
*MSH2* variants	No variations	No variations	No variations	No variations	No variations	No variations	No variations	c.2754G > A	No variations	No variations	No variations	c.1241 T > G	No variations
Disease	No	IBD	No	No	Anemia	No	No	n/a	Anemia	No	Anemia & diabetes	No	Anemia & IBD
FH	neg	pos	neg	neg	pos	pos^a^	pos^b^	pos	pos	pos	pos	pos	pos^c^
Metastasis	Yes(2005‐liver)	No	No	No	No	No	No	No	No	No	No	(2010‐lungs)	No

Comparison of clinicopathological and demographic data												
Patients ID	sf/27	sd/28	As/1	hd/13	ps/24	hm/25	Ah/5	Bh/7	Bg/9	Fm/12	Mk/18	Sm/26
Sex	F	F	M	M	F	M	M	F	F	F	M	M
Tumor location	Rectum‐Left‐	Ascending colon‐Right‐	Rectum‐Left‐	Splenic flexure‐Left‐	Descending and sigmoid‐Left‐	Rectum‐Left‐	Ascending colon‐Right‐	Rectum‐Left‐	Ascending‐Right‐	Secum‐Right‐	Ascending colon‐Right‐	Ascending colon‐Right‐
Differentiation	Well	Well	Poor	Well	Well	Well	Well	Well	Moderate	Well	Well	Poor
TNM	M0/N1/T1	M0/N0/T2	M0/N1/T3	n/a	M1/N0/T3	M0/N0/T3	n/a	M0/N0/T3	M0/N1/T3	M0/N0/T3	M0/N0/T3	n/a
Stage	IIIA	I	IIIB	I	IV	IIA	II	IIA	IIIB	IIA	IIA	II
MSI status	MSI‐H	MSI‐H	MSI‐H	MSI‐H	MSI‐H	MSI‐H	MSI‐H	MSI‐H	MSI‐H	MSI‐H	MSI‐H	MSI‐H
Diagnosis age	40	75	27	24	43	30	45	38	37	41	40	52
Live/death	D	L	L	L	L	L	L	L	L	D	L	L
*MLH1* variants	c.407A > T c.826A > T c.423A > C	c.1612 T > G c.1592 T > G	No variations	No variations	c.1592 T > G	No variations	c.889G > A	c.1592 T > G	c.1612 T > G c.1592 T > G	c.1612 T > G c.1592 T > G	c.412C > A	c.1612 T > G c.1592 T > G
*MSH2* variants	No variations	c.2701G > A c.2704G > A	No variations	No variations	c.2599G > T	No variations	No variations	c.2613G > A	No variations	c.2524G > A	No variations	c.842C > G
Disease	No	No	No	No	No	No	No	Diabetes	No	No	No	No
FH	pos	neg	pos^d^	pos	pos^e^	neg	neg	pos	pos	neg	neg	pos
Metastasis	No	No	No	n/a	Yes liver	n/a	n/a	n/a	n/a	n/a	n/a	n/a

^a^ Paternal cousin‐brain tumor.

^b^ Paternal aunts and uncles‐CRC/father‐GC.

^c^ Father‐prostate cancer/maternal aunt‐brain tumor maternal aunt breast cancer/maternal cousin‐leukemia paternal cousin‐CRC.

^d^ Sister‐endometrial cancer/father, sister, paternal aunt, maternal cousin, and paternal grandmother‐CRC.

^e^ Brother, father, two paternal aunts, paternal uncle, paternal cousin‐CRC/brother‐brain tumor.

### 2.4 Statistical analysis

The data obtained were analyzed using the Statistical Package for the Social Sciences (SPSS) 21.0 statistical package (Chicago, IL, USA). Data were presented as mean ± standard deviation. Variables, based on the data, were compared by Chi‐square or Fisher's exact tests and a non‐parametric test (Mann–Whitney or Kruskal Wallis).

The Chi‐square test was used to determine the relationships among the clinicopathologic factors. Statistical significance was defined as two‐tailed *P* < .05.

## 3 RESULTS

All patients had undergone MSI tests, and only MSI‐H patients were included in the study (Table 2). To analyze our study population, we concentrated on three primary panels: demographics (age of diagnosis and gender), clinicopathologic factors (tumor location, tumor differentiation, stage, MSI status, etc.), and the features of variants themselves (Pathogenicity, evaluating the affected domains and interacted, repeating variants, etc.). Our results demonstrated that from 25 studied patients, 22 cases, comprising 10 males (45.4%) and 12 females (54.5%), showed at least one variation in the exonic regions of the *MLH1* gene, while 3 cases showed no variants in the *MLH1* (Table 2). Concerning the tumor location among our studied population, 14/25 (56%) patients were left‐sided, and 11/25 (44%) were right‐sided. It is also worth mentioning left‐sided tumors were more common among males rather than females (males: 5 right‐sided tumors and eight left‐sided tumors/females: 6 right‐sided tumors and six left‐sided tumors) (regardless of variant detection) (Table 2). Toward The *MSH2* gene, seven patients revealed at least one variation in the exonic regions of the *MSH2*. Of them, one carrier out of 7 was male (14.2%), and six carriers out of 7 were female (85.8%). However, 18 cases showed no changes in the *MSH2* gene (Table 2). The chi‐square test was used to distinguish differences between sex and *MLH1,* and *MSH2* variations. A significant *p*‐value was observed between *MSH2* and sex in this survey (*p*‐value <.021, Mean ± SD: 1.72 ± 0.458), but no significant change was observed in *MLH1* and gender (*p*‐value <.08, Mean ± SD: 1.12 ± 0.332). In our study population, male patients showed a lower mean age (42.2) at CRC diagnosis compared to female patients (44.16). Moreover, in both sexes, patients harboring variants in the *MLH1* gene were diagnosed at lower ages than those carrying variations in the *MSH2* gene (Table 2). Interestingly, there is a significant *p*‐value (<.016, Mean ± SD: 41.56 ± 11.15) between younger ages and variation observation in *MLH1*, but no significant data was observed between *MSH2* variations and included patients age (*p*‐value <.187). Regarding the features of the variants (regardless of their repeats in our studied population), 21 specific variants were detected in the exonic regions of the *MLH1* and *MSH2* genes. Our finding revealed that variants in the *MLH1* gene (regardless of their pathogenicity) are more common than *MSH2* gene (Table 2). The *MLH1* gene contains 13 exonic variations, with 30.7% in exon 5, 15.4% in exon 11, 15.4% in exon 14, and 15.4% in exon 18. Exons 8, 10, and 17 had a 7.7% occurrence of variants, whereas exon 14 had the highest frequency of recurring variants (Tables 2 and 3). When it came to the incidence of individual domain variations, the N‐terminal domain had the highest rate of occurrence (61.5%), followed by the C‐terminal domain (38.5%) (Table 3). However, when repeats of variants were also considered, the C‐terminal domain showed the upper hand (Tables 2 and 3). As mentioned earlier, domains and regions of the *MLH1* gene interact with other elements of the MMR system. 30.8% of specific variants were located in a region that has interaction with MutS homologs, 30.8% had interaction with no elements, 23% had interaction with PMS1/MLH3/PMS2, and 15.4% of these 13 specific variants had interaction with PMS1/MLH3/PMS2 among with Exonuclease 1 (EXO1) regions (Table 3). Regarding the frequency, PMS1/MLH3/PMS2 regions were most affected (Tables 2 and 3). Our unique detected variants in the *MLH1* gene were mostly missense type, with 92% frequency and 8% of the variants being synonymous (Table 1S). Among this portion, we found four variants, including *MLH1*: c.655A > G (p.I219V), c.889G > A (p.E297K), c.977 T > C (p.V326A), c.1612 T > G (p.W538G), and c.2002G > A (p.E668K) which were previously reported in studies.^22^, ^23^, ^24^, ^25^, ^26^ There was also a variant (*MLH1* c.1969A > T (p.I657F)) that was available in the dbSNP database. Regarding this variant, VUS (variant of uncertain significance) prediction was the consequence, according to our observations, but the clinical significance for this variant in this database was not available.^27^ It is also worth mentioning that high‐frequency repeats of some mentioned variants were seen in the sequenced *MLH1* gene exons that are discussed in detail in the following.

**TABLE 3:** Pathogenicity and structural features of *MLH1* variants.

Variants	ACMG Guideline predicts	Amino acid change	Charge changing	Polarity changing	Exon	Regions affected by variants
^a^ **c.394G > A** **p.D132N**	PM2‐PM5‐PP1‐PP3‐PP4 (VUS)	Aspartate to aspargin	Yes/negative to neutral	No/polar to polar	5	N‐terminal domain
^a^ **c.407A > T** **p.K136A**	PM2‐PP1‐PP3‐PP4 (VUS)	Lysine to alanine	Yes/positive to neutral	Yes/polar to non‐polar	5	N‐terminal domain
^a^ **c.412C > A** **p.P138T**	PM2 (VUS)	Proline to threonine	No/neutral to neutral	Yes/non‐polar to polar	5	N‐terminal domain
^a^ **c.423A > C** **p.P141P**	PM2‐BP4‐BP7 (Likely benign)	No/same amino acid	No/same amino acid	No/same amino acid	5	N‐terminal domain
c.655A > G (rs1799977) p.I219V (Pineda et al.)	BA1‐BS3‐BP4 (Benign)	Isoleucine to valine	No/neutral to neutral	No/non‐polar to non‐polar	8	N‐terminal domain Muts homologs interaction
^a^ **c.826A > T** **p.I276L**	PP1‐PM2‐PM5‐PP4 (VUS)	Isoleucine to leucine	No/neutral to neutral	No/non‐polar to non‐polar	10	N‐terminal domain Muts homologs interaction
c.889G > A (rs63750736) p.E297K Urso E. et al.,	PM2‐PP3 (VUS)	Glutamate to lysine	Yes/negative to positive	No/polar to polar	11	N‐terminal domain Muts homologs interaction
c.977 T > C (rs63751049) p.V326A (Park KJ et. al)	PP1‐PP3‐PP4‐PM2 (Benign due to reported data)	Valine to alanine	No/neutral to neutral	No/non‐polar to non‐polar	11	N‐terminal domain Muts homologs interaction
^a^ **c.1592 T > G** **p.V531G**	PM2‐PP3‐PP4‐PP1 (VUS)	Valine to glycine	No/neutral to neutral	No/non‐polar to non‐polar	14	C‐terminal domain PMS2/MLH3/PMS1 and EXO interaction
c.1612 T > G (rs1559575214) p.W538G ^48^	PM2‐PP3‐PP4‐PP1 (VUS)	Tryptophan to glycine	No/neutral to neutral	No/non‐polar to non‐polar	14	C‐terminal domain PMS2/MLH3/PMS1 and EXO interaction
c.1969A > T (rs765363859) p.I657F	PM2‐PP3 (VUS)	Isoleucine to phenylalanine	No/neutral to neutral	No/non‐polar to non‐polar	17	C‐terminal domain PMS2/MLH3/PMS1
c.2002G > A (rs63750292) p.E668K Ali H et al.	PM2‐PP3‐PP4‐PP1 (VUS)	Glutamate to lysine	Yes/negative to positive	No/polar to polar	18	C‐terminal domain PMS2/MLH3/PMS1
^a^ **c.2005G > A** **p.E669K**	PM2‐PP3‐PP4‐PP1 (VUS)	Glutamate to lysine	Yes/negative to positive	No/polar to polar	18	C‐terminal domain PMS2/MLH3/PMS1

^a^ Novel variants are written in bold.

Highly‐frequent repeats among these nucleotide changes in the *MSH2* gene were not seen (as mentioned earlier, a different pattern regarding the occurrence of the variants was seen in the *MLH1* gene). Moreover, eight exonic variants have been found in the *MSH2* gene consisting of 62.5% in exon 15, 12.5% in exon 5, 12.5% in exon 7, and 12.5% in exon 16 (Table 4).

**TABLE 4:** Pathogenicity and structural features of *MSH2* variants.

Variants	ACMG	Amino acid change	Charge changing	Polarity changing	Exon	Regions affected by variants
c.842C > G p.S281Ter (Zhu F et. Al) rs63749991	PVS1‐PM2‐PP1‐PP4 (Pathogen)	Serine to stop codon	No amino acid	No amino acid	5	7
c.1241 T > G p. L414R rs587779078	PM2‐PP3‐PP4‐PP1‐PM5 (VUS)	Leucine to arginine	Yes/neutral to negative	Yes/non‐polar to polar	7	Lever domain MSH6/MSH3 interaction
c.2524G > A p. E842K	PM2‐PM1 (VUS)	Glutamate to lysine	Yes/negative to positive	No/polar to polar	15	ATPase domain
^**a**^ **c.2599G > T** **p. E867Ter**	PVS1‐PM2‐PP4‐PP1 (Pathogen)	Glutamate to stop codon	No amino acid	No amino acid	15	Helix‐turn‐helix domain
^a^ **c.2613G > A** **p. K871K**	BP7‐PP4‐PP1 (VUS)	No/same amino acid	No/same amino acid	No/same amino acid	15	Helix‐turn‐helix domain
^a^ **c.2701G > A** **p. E901K**	PM2 (VUS)	Glutamate to lysine	Yes/negative to positive	No/polar to polar	15	Helix‐turn‐helix domain MSH6/MSH3 interaction
^a^ **c.2704G > A** **p. E902K**	PM2 (VUS)	Glutamate to lysine	Yes/negative to positive	No/polar to polar	15	Helix‐turn‐helix domain MSH6/MSH3 interaction
^a^ **c.2754G > A** **p.K918K**	BP7‐PP4‐PP1 (VUS)	No/same amino acid	No/same amino acid	No/same amino acid	16	Helix‐turn‐helix domain MSH6/MSH3 interaction

^a^ Novel variants are written in bold.

Affected domains, regarding the occurrence and the presence of the variants, were studied as well. 62% of 8 specific variants were observed in the helix‐turn‐helix domain, 12.5% in the connector domain, 12.5% in the lever domain, and 12.5% in the ATPase domain (Table 4). Respecting the regions of the *MSH2* gene that interact with other components of the MMR system, it was observed that 50% of the eight detected variants were located in a region interacting with MSH6/MSH3, while 50% had no interaction with known elements (Table 4). Concerning the type of nucleotide changes in *MSH2*, 50% of variants were missense, 25% non‐sense, and 25% synonymous (Table 2S), and one variant among the found *MSH2* variants (*MSH2* c.842C > G (p.S281Ter)) was previously reported in a study.^28^ Also, two variants (*MSH2*: c.1241 T > G (p. L414R) and c.2524G > A (p. E842K)) were available on the NCBI database, and no pathogenicity (like our observations) were detected as their consequence.^29^

As the observed variants were frequent in our study population, we categorized them into three main groups, including frequent variants, suspected single nucleotide variants (SNVs), and patients with no variants.

### 3.1 Highly‐frequent variants in the *MLH1* gene

**FIGURE 1:**
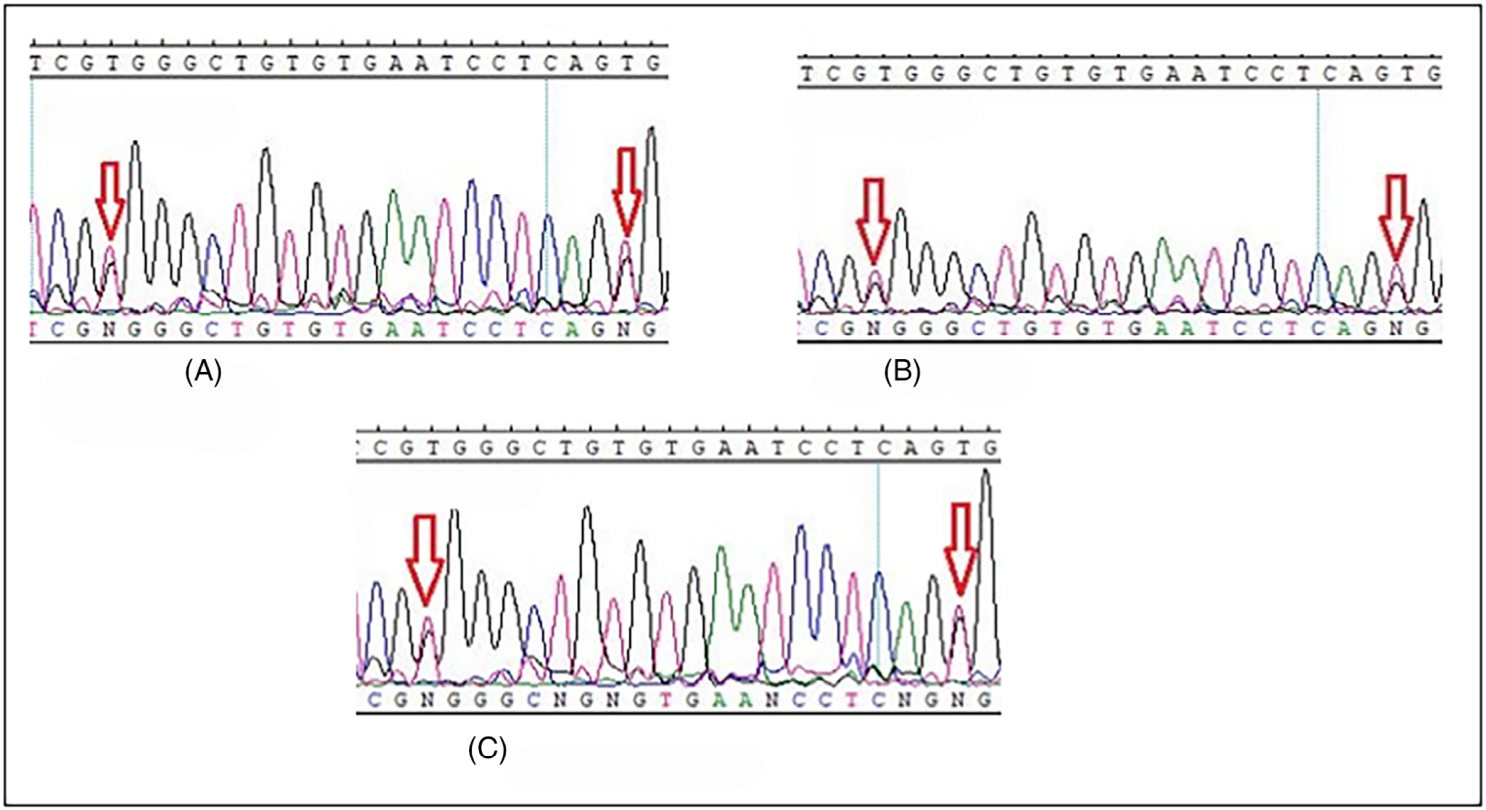
Sequence diagram of the *MLH1*: c.1612T>G (p.W538G) and c.1592T>G (p.V531G) variants in three different patients. The red arrows indicate the position of each mutation on the forward strand. (A) Patient Fm/12 (B) Patient Lr/17 (C) Patient Af/3.

**FIGURE 2:**
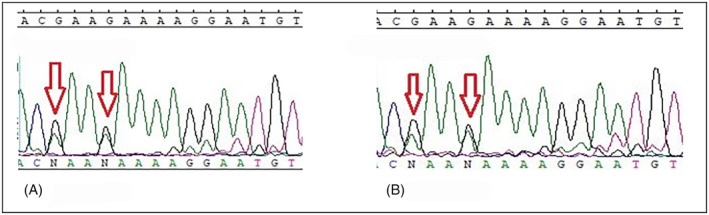
Sequence diagram of the *MLH1*: c.2002G>A (p.E668K) and c.2005G>A (p.E669K) in two different patients. The red arrows indicate the position of each mutation on the forward strand. (A) Patient Mh/23 (B) Patient Mh/20.

*MLH1*: c.1612 T > G (p.W538G) and c.1592 T > G (p.V531G) were found in pairs in 13 different cases (*MLH1* c.1592 T > G (p.V531G) was also found in two patients alone) and with lower frequency *MLH1*: c.2002G > A (p.E668K) and c.2005G > A (p.E669K) were found in 5 different cases (Table 2). All these four variants occur in the C‐terminal domain of the *MLH1* gene, and this region interacts with PMS1/MLH3/PMS2 elements, which play a critical role in the MMR system (Table 3). As mentioned above, these variants affect most patients. To avoid repetition, we showed limited sequencing results in Figures 1A–C and 2A, B (other results are also available but not shown). Also, patients harboring these four variants did not show specific features to verify the exact demographic and pathobiological effects that result from such SNV. However, the interesting finding was that tumors in stage IV were not a common phenomenon among these patients. The repetition of these variants among our population deprived us of the exact genotype/phenotype conclusion that can be resulted from the occurrence of mentioned variants.

(It is also worth mentioning that patients Ay/6, and Mh/23, who were *MLH1*: c.1612 T > G (p.W538G) and c.1592 T > G (p.V531G) carriers, had IBD as well. Due to the findings, this occurrence does not happen accidentally and has some records, too (Further evaluations are included in the discussion section.)

### 3.2 Suspected SNVs

#### 3.2.1 *MSH2* c.842C>G (p. S281Ter)

**FIGURE 3:**
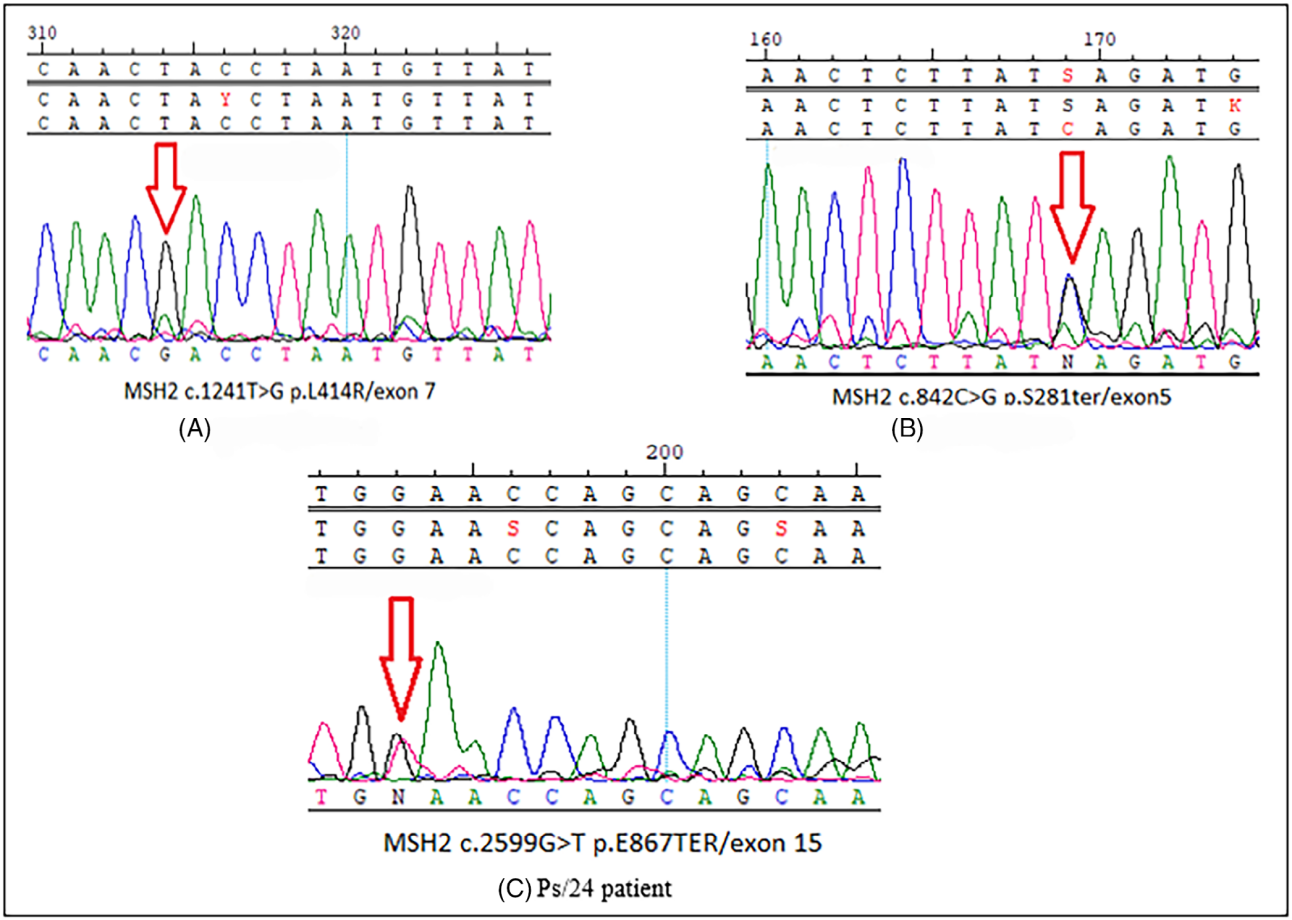
Sequence diagrams of the c.1241T>G p.L414R (A), c.842C>G p.S281Ter (B), c.2599G>T p.E867Ter (C), the suspected SNVs in the *MSH2* gene. The red arrows indicate the position of each mutation on the forward strand. (A) *MSH2* c.1241T>G (p.L414R)/Exon 7, Patient Mm/21 (B) *MSH2* c.842C>G (p.S281Ter)/Exon 5, Patient Sm/26 (C) *MSH2* c.2599G>T (p.E867Ter)/Exon 15, Patient Ps/24.

This variant is located in the fifth exon of the *MSH2* gene (Figure 3B) and on the connector domain of the protein (Table 4). The tumor location of this carrier (SM/26) was right‐sided, his family history was positive (Supplementary Figure 2) (Table 2), and the pathogenicity of this non‐sense variant is reported not only in three different sources (including CADD, Provean online software, and Clinvar database [Supplementary Table 2]) but also has been demonstrated in experimental studies too (Table 4).

#### 3.2.2 *MSH2* c.2599G>T (p. E867Ter)

This variant is located in the 15th exon of the *MSH2* gene (Figure 3C) and in the helix‐turn‐helix domain. A liver metastasis was observed in the patient who harbors this variant, and this case's primary tumor was left‐sided (Table 4). Concerning the patient's family history (Supplementary Figure 3), 1st‐ and 2nd‐degree relatives were affected. Moreover, based on revised Bethesda criteria, the variant leads to a common form of LS pathogenicity, consisting of the frequent occurrence of Lynch‐related diseases (including CRC) in 1st and 2nd‐degree relatives. The *MSH2* c.2599G > T (p. E867Ter) is a nonsense variation in the exonic region, which causes a truncated protein. This fact, along with the phenotypic features of the disease, guided us to report this novel variant as a pathogenic variant based on the S. Richards et al. standard.^21^

#### 3.2.3 *MSH2* c.1241T>G (p.L414R)

This variant is located in the 7th exon of the *MSH2* gene that affects the lever domain of the protein, which interacts with MSH6/MSH3. Tumorigenesis was present in the carrier and was found on the right side of the colon leading to lung metastases in this patient (Table 2). This missense variant results in a leucine‐to‐arginine substitution that causes shifts in the polarity and charge of the protein (Table 4). Software and databases, including Polyphen, Meta LR, and REVEL, count this SNV as a damaging variant (Supplementary Table 2). Considering that LS is an autosomal dominant disease, the presence of a homozygous variant is rare among patients with sLS. However, we detected a homozygous variants one patient with positive family history (Supplementary Figure 4) in our studied population (Figure 3A).

### 3.3 sLS patients without variants on either *MLH1* or *MSH2* exonic regions

Although growing evidence reveals that germline variations in *MLH1* and *MSH2* genes are likely responsible for around 60%–70% of LS,^13^ we have found three probands (As/1, Bg/9, Hm/25) without variants in either *MLH1* or *MSH2* genes and the primary tumors found on these three unique cases were interestingly left‐sided (Table 2). Since all three cases were under the age of 30 with positive family history and MSI‐H, these patients were highly suspected of LS based on revised Bethesda guidelines.

## 4 DISCUSSION

In the present study, we mainly focused on the spectrum, frequency, and distribution of the pathogenic *MSH2* and *MLH1* variants among cases with MSI‐H that met the revised Bethesda criteria. Based on ACMG guidelines, the overall ratio of detected pathogenic variants in the *MLH1* and *MSH2* genes was 23.8% (Tables 3 and 4). Regarding the tumor location, as mentioned in the result, interestingly, the left‐sided tumors were more frequent among the studied population, which is in contrast with the usual form of LS^30^ (Table 2). The mentioned phenomenon is not a novel occurrence; it has been documented in the Chinese population as well and it was considered to be a population related feature.^30^, ^31^ It is worth mentioning that pathogenic changes are observed more frequently in the *MLH1* variants than in the *MSH2* (in Supplementary Figure 1, the occurrence regions of the variants are depicted). As far as it is understood, the MMR process consists of multiple genes and proteins in its way of functioning. So, the pathogenic variants do not necessarily appear in one or two specific genes like *MLH1* or *MSH2*, and even the MSI status can be stable among affected people due to the absence of Knudson's second hit, and further tumor‐specified features will not happen as well, but the pathogen variant can still be passed down to the next generation. In the study of Olkinuora A et al., It was concluded that the analysis of *MLH1* and *MSH2* genes is not fully trusted, and therefore multi‐gene panels and protein status tests should be considered for all genes.^32^ According to our data, females have a higher percentage of *MLH1* and *MSH2* carriers, which is in line with Muller. P. et al., However, the difference in frequency between these two populations is statistically significant. In our study, the diagnosis age of CRC has been considered as well. The mean age of *MLH1* (*n* = 22) and *MSH2* (*n* = 7) carriers was 43.5 and 46.28 years old, respectively. The disparity between these statistics and those in the Muller et al., study could be attributed to timely referral or screening guidelines, but in general, in both studies, the diagnosis of CRC among *MLH1* carriers was lower.^33^ However, the pathogenicity fingerprint among our patients and other reported cases was not always consistent, as in the study conducted by Mangold et al. in the German population, in which *MSH2* pathogenic carriers were shown to have the upper hand, which is utterly contrary to our findings.^34^

In the present study, 21 specific variants were found in the *MLH1* and *MSH2* genes, and of these, pathogenicity was mostly available among *MLH1* variants. This is in line with the findings of Valentin et al., who studied the South American population. Valentin et al. discovered that variants (pathogenic or non‐pathogenic) in their *MLH1* and *MSH2* genes occur in regions interacting with other proteins in the MMR system. It was concluded that these multiple variants, which happen in interacted regions, may cause polygenic damage to the performance of the MMR system, and other genes involved in this process need to be studied as well.^35^ The location of the detected variants in our findings took place in some particular domains that further have specific interactions at the protein level. Regarding our study, 5 out of 13 specific variants, including four of the most frequent variants in the *MLH1* gene, including *MLH1*: c.1612 T > G (p.W538G), c.1592 T > G (p.V531G), c.2005G > A (p.E669K), and c.2002G > A (p.E668K) happen in the c‐terminal domain of the gene that interacts with PMS2/MLH3/PMS1. According to a study conducted by Wijnen et al. on Danish and Italian patients, the major disease‐causing variations in individuals are clustered in exons 15 and 16. However, according to our findings, no variations in exons 15 and 16 were discovered, which is in contrast to Wijnen et al. findings. However, it should be noted that exons 15 and 16 are located in the gene's c‐terminal domain, which interacts with PMS2/MLH3/PMS1, implying that the region of occurrence of the variants and the state of their interaction are strikingly similar.^36^ The *MSH2* c.2599G > T (p.Glu867Ter) variant is a non‐sense variant that may damage the overall state of the protein. This variant occurred once in Ps/24 patient (Table 2). He had a diagnosis of stage IV CRC with the presence of tumors in the sigmoid region and descending colon along with metastasis in his liver. His family history is similarly positive, with CRC identified in his brother, father, two aunts, uncles, and a cousin, as well as a brain tumor in his brother (Supplementary Figure 3). This variant was considered pathogenic according to ACMG classification.^37^ Reliable data are available For determining the pathogenicity of this variant in our study, including the presence of a non‐sense variant, positive family history, and so forth^38^ (Table 4). According to all mentioned facts, this novel variant can be reported as a pathogenic one due to ACMG guidelines.

All of the variants observed in this study were heterozygous. However, in the Mm/21 case, we detected a homozygous variant *MSH2* c.1241 T > G (p.L414R). Homozygosity has been also reported in other autosomal dominant syndromes linked to CRC, such as bi‐allelic changes in the *APC* gene in FAP syndrome.^39^. One interesting fact about this type of nucleotide change in LS cases is its rarity among carriers since LS is an autosomal dominant disease and the heterozygous variant is the most common form of occurrence.^40^, ^41^ Homozygous carriers demonstrate certain characteristics, such as early detection of leukemia, lymphoma, Cafe'‐au‐Lait spots, brain tumors, and a higher susceptibility to cancer (that is mainly known as constitutional mismatch repair deficiency, CMMRD), which can be detected at a younger age than other cases.^42^ In some cases, these characteristics differ, and individuals develop conditions such as a high prevalence of polyposis and various malignancies at younger ages.^43^ This phenomenon was discovered in European and Asian populations and in recent years, there has been an increase in the number of studies and case reports on this occurrence. As The occurrence of the homozygous variants does not necessarily follow a particular pattern, so almost any sequence of the MMR genes can be a probable region for their presence.^44^, ^45^, ^46^

The carrier of *MSH2* c.1241 T > G (p.L414R) variant (classified as a VUS based on ACMG guidelines) is a 48‐years‐old female with a relatively long life compared to other reported cases of homozygous variants. It can be concluded that the presence of a homozygous variant in an individual does not necessarily increase the risk of mortality. This finding confirms the contradiction of a 2005 study in Germany by Muller et al.^47^ The involvement of CRC, in this case, has been identified at the age of 48, indicating a late diagnosis. Metastasis, has also been observed, suggesting an advanced stage of the disease at the time of diagnosis. It is worth considering that the person may have experienced a mild form of cancer at an early age. Studies conducted by J. Muller et al. have shown that this particular issue is commonly observed in carriers of the homozygous variant. The lack of noticeable changes in the patient's quality of life, coupled with the absence of apparent symptoms, may have contributed to her decision not to seek medical help. Although an obvious positive family history for this case was seen (Supplementary Figure 4), due to the lack of screening or follow‐up processes for this family, the person may not have been aware of the need for regular cancer screening programs. Resultantly, the person was diagnosed with a high stage of malignancy at the age of 48.

*MSH2* c.842C > G (p. S281Ter) is a nonsense variation reported as damaging based on various software and databases (Supplementary Table 2) and classified as pathogenic based on ACMG guidelines (Table 4). In our study, this variant has been detected in a 52 years old man with tumor stage II, and positive family history. Unfortunately, additional information about his history was not available and a reliable conclusion about this variant did not arise. Formerly, this variant was detected in a Chinese population, and it had been reported as a pathogenic variant.^28^

Several missense variants, including *MLH1* c.1612 T > G (p.W538G), c.1592 T > G (p.V531G), c.2005G > A (p.E669K) and c.2002G > A (p.E668K) were frequently detected in the *MLH1* gene in carriers. Since these variants were present with high recurrence in pairs or individually (*MLH1*: c.1612 T > G (p.W538G) and c.1592 T > G (p.V531G) were usually detected in pairs), it is crucial to address these recurrent changes separately. Two variants of *MLH1*: c.2002G > A (p.E668K) and c.1612 T > G (p.W538G), have been reported as pathogenic in one and three software indices (Supplementary Table 1), respectively. According to our available evidence, the *MLH1* c.1612 T > G (p.W538G) variant could be reported as VUS based on ACMG guidelines; it is worth mentioning that this variant was first submitted by InSIGHT^48^ and conflictions over the pathogenicity of this specific variant still exist. In the Parc et al. study^49^ it is reported as de novo in supplementary (it should be mentioned that the frequency of the occurrence of this variant is evaluated in the French population, and it was not as frequent as ours) but as we saw on InSIGHT it was previously reported as VUS by Thompson et al.^24^ As mentioned, due to the repetitive occurrence of these variants, and complications that they represent, a reliable conclusion that could link demographic, clinicopathological, and so forth. together was not possible, but the only common variable among these cases was IBD disease. Patients Ay/6 and Mh/23 had IBD (Table 2). This has been reported before, and the findings of Derikx et al. and Hahn et al. confirm it. The occurrence of these two diseases simultaneously shows an increased risk of CRC in lower median age that should be measured in a more significant population in future studies.^50^, ^51^ Based on a different point of view, as these variants were frequently observed within our studied population, it led us to suspect the existence of the founder effect which is deemed essential in understanding this phenomenon.^52^ In relation to this occurrence, we did not find any meaningful result regarding this variation in other races and regions, so this occurrence should be considered in the future studies of Iranian population in a more profound manner which needs special techniques and tests like next generation sequencing (NGS) that provide more information at a lower cost and in less time.^53^, ^54^

Three of the carriers we examined, did not show any variants in the exons of the *MLH1* and *MSH2* genes. It is generally hypothesized that the main cause of this phenomenon could be *MLH1*‐promotor methylation followed by a *BRAF* pathogenic variant or even a chromosomal instability which explains our inability to detect any germline variant in their exons. The challenging part of these three patients is their MSI status. Although it has been seen frequently in LS, it is not always related to this syndrome and it can also be seen in 10%–15% of sporadic CRC cases.^55^ In these cases, some dark aspects still remain unanswered ranging from early age tumor detection to positive family history which needs a more comprehensive look to achieve a reliable answer for their current complicated situation that unfortunately is not currently achievable.

The value of studying the variations detected in LS has recently been assumed to be beneficial, and it has become a reliable approach to understanding this multi‐factor syndrome.^56^ According to the mentioned hypothesis, evaluations of the variants in various aspects, such as pathogenicity, structure of the resulting amino acids, clinicopathological features of the carriers, and demographical data, must be conducted in different populations. In this study, our aim was to present and evaluate these data in a single project to achieve a perspective regarding this syndrome and its features in our population. As mentioned in the context, we detected a very rare phenomenon in our study population, from the detection of a high number of carriers to homozygous variants (in a small volume of cases). The strength of this compact study is its ability to raise awareness about the specific characteristics of this inherited disease to alert experts and enhance genetic consulting.

As an example, it is important to mention that one‐third of LS cases do not exhibit any genetic variation. In such cases, techniques like investigating large deletions or duplications aka large genomic rearrangements^57^ which were unfortunately not accessible during our study and are considered as one of our limitations, offer significant assistance. Besides mentioned ones, there were other limitations in our study too. These limitations can be overcome by increasing the study population, using more up‐to‐date methods, and conducting more comprehensive studies such as meta‐analyses. As a new perspective, despite the study of variations, the gene‐specific guidelines are getting more pervasive with the introduction of the European Hereditary Tumor Group, which needs updated methods like whole genome sequencing.^58^, ^59^

## 5 CONCLUSION

The proportion of genetic variants in both genes was higher in women in our study, and most of the significant variants in both sexes were missense. The major *MLH1* gene variants were found in the C‐terminal domain, while the *MSH2* gene variants occurred in the helix‐turn‐helix domain. Interestingly, as a characteristic of our studied population, we observed repetitive variations among our cases, which requires further investigation to potentially identify a new founder variant and its certain pathogenicity. In relation to this disease and our findings, due to the presence of suspected cases with no variants in both *MLH1* and *MSH2* genes, it is recommended that complementary tests be considered to avoid any risks and not to let any hereditary roots left uninvestigated. To lower this risk, current two‐gene study model focusing on *MLH1* and *MSH2* genes should be revised to a gene panel model including all genes in the MMR system and genes involved in this pathway. Additionally, investigating other susceptible genes by using cutting‐edge techniques such as NGS and informatics in larger population studies can contribute to this investigation, and specific demographic factors, such as the higher proportion of female carriers, should also be taken into account.

## AUTHOR CONTRIBUTIONS

**Seyed Mohsen Mirabdolhosseini:** Conceptualization (equal); investigation (lead); methodology (equal); writing – original draft (lead). **Mohammad Yaghoob Taleghani:** Conceptualization (equal); formal analysis (equal); methodology (equal). **Leili Rejali:** Conceptualization (equal); formal analysis (equal); writing – review and editing (equal). **Hossein Sadeghi:** Data curation (equal); validation (equal); writing – review and editing (equal). **Nayeralsadat Fatemi:** Formal analysis (equal); software (lead); writing – review and editing (equal). **Mehdi Tavallaei:** Data curation (equal); validation (equal). **Amin Famil Meyari:** Formal analysis (equal); methodology (equal). **Narges Saeidi:** Formal analysis (equal); methodology (equal). **Pardis Ketabi Moghadam:** Validation (equal); writing – review and editing (equal). **Amir Sadeghi:** Resources (equal). **Hamid Asadzadeh Aghdaei:** Funding acquisition (equal); resources (equal). **Mohammad Reza Zali:** Funding acquisition (equal). **Ehsan Nazemalhosseini Mojarad:** Conceptualization (equal); data curation (equal); project administration (lead); supervision (lead); writing – original draft (equal); writing – review and editing (equal).

## FUNDING INFORMATION

This project was supported entirely and funded by the Gastroenterology and Liver Diseases Research Center, Research Institute for Gastroenterology and Liver Diseases, Shahid Beheshti University of Medical Sciences, and the Medical Ethical Committee of the RCGLD, with Ethics No. IR.SBMU.RIGLD.REC.1396.947.

## CONFLICT OF INTEREST STATEMENT

The authors declare no conflicts of interest.

## ETHICS STATEMENT

All procedures performed in studies involving human participants were in accordance with the ethical standards of the institutional and/or national research committee and with the 1964 Helsinki declaration and its later amendments or comparable ethical standards.

## INFORMED CONSENT

Informed consent was obtained from all participants included in the study.

## Supporting information


**Supplementary Figure 1.** The schematic structure of the *MLH1* and *MSH2* genes along with the occurrence of the observed specific variants: (The schematic structure of the genes is obtained from the noticed reference.^59^

**Supplementary Figure 2.** The pedigree of SM/26 patient.

**Supplementary Figure 3.** The pedigree of PS/24 patient.

**Supplementary Figure 4.** The pedigree of MM/21 patient.

**Supplementary Table 1.** In silico prediction of the *MLH1* variants.
**Supplementary Table 2.** In silico prediction of the *MSH2* variants.

## Data Availability

All presented data in this study are available on request from the corresponding author (Ehsan Nazemalhosseini Mojarad). The data are not publicly available due to privacy or ethical restrictions.
