# Supplementary figures and images for: Rare single‐nucleotide variants of MLH1 and MSH2 genes in patients with Lynch syndrome
Source: Cancer Rep (Hoboken). 2023 Nov 2;7(1):e1930. doi: 10.1002/cnr2.1930 (PMC10809271; doi:10.1002/cnr2.1930)

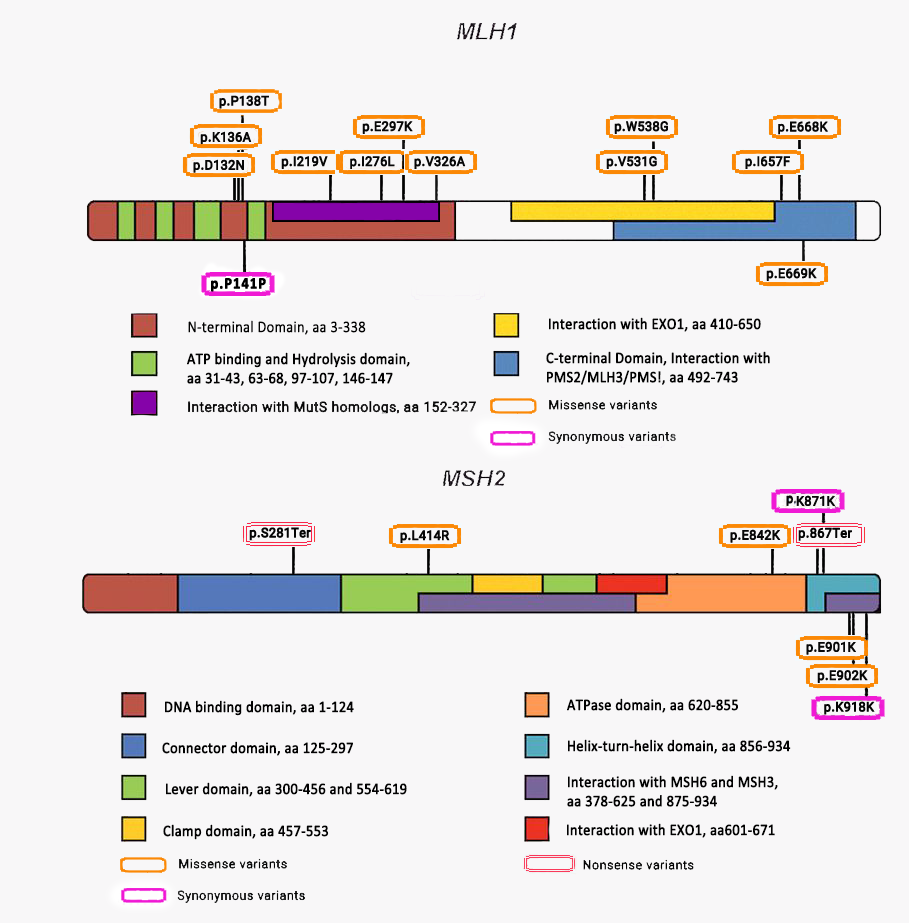

Supplement: Supplementary file 1 — Supplementary Figure 1. The schematic structure of the MLH1 and MSH2 genes along with the occurrence of the observed specific variants: (The schematic structure of the genes is obtained from the noticed reference.59 [file CNR2-7-e1930-s003.tif]

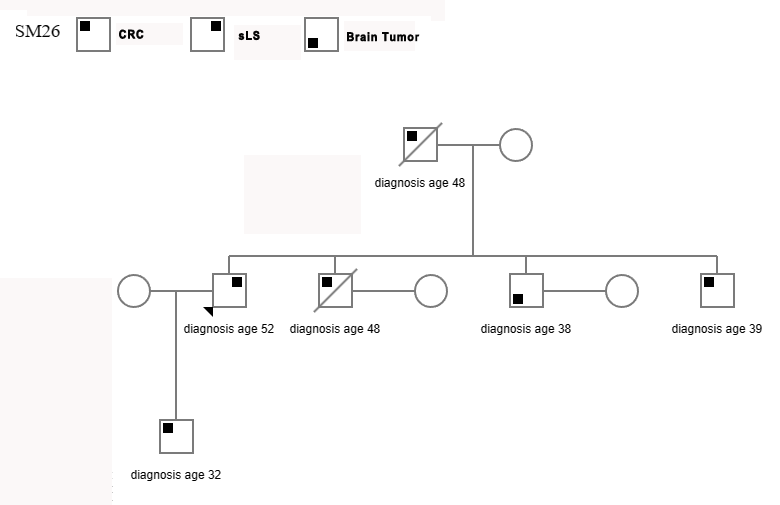

Supplement: Supplementary file 2 — Supplementary Figure 2. The pedigree of SM/26 patient. [file CNR2-7-e1930-s002.png]

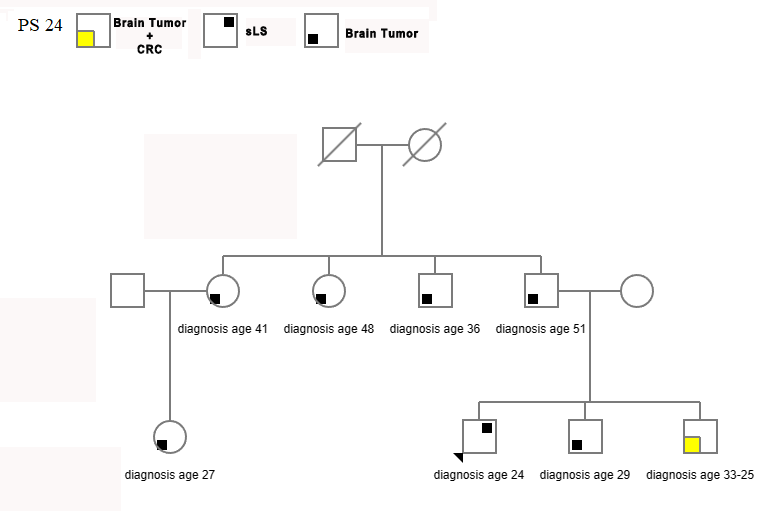

Supplement: Supplementary file 3 — Supplementary Figure 3. The pedigree of PS/24 patient. [file CNR2-7-e1930-s004.png]

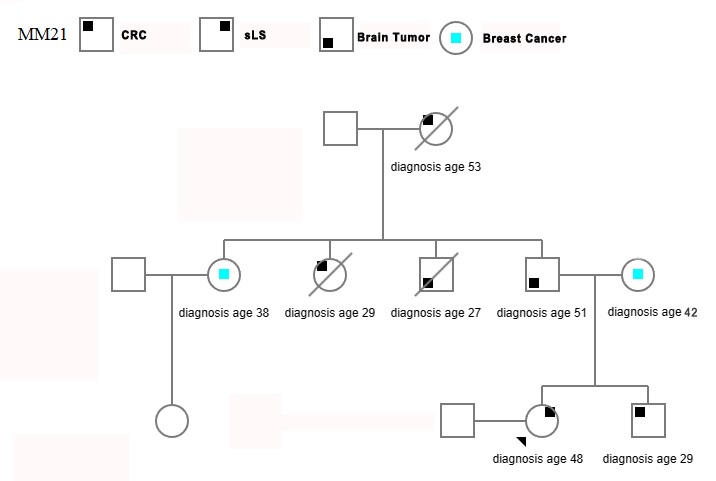

Supplement: Supplementary file 4 — Supplementary Figure 4. The pedigree of MM/21 patient. [file CNR2-7-e1930-s001.jpg]
