# Supplementary material for: Rare single‐nucleotide variants of MLH1 and MSH2 genes in patients with Lynch syndrome
Source: Cancer Rep (Hoboken). 2023 Nov 2;7(1):e1930. doi: 10.1002/cnr2.1930 (PMC10809271; doi:10.1002/cnr2.1930)
Supplement: Supplementary file 5 — Supplementary Table 1. In silico prediction of the MLH1 variants. Supplementary Table 2. In silico prediction of the MSH2 variants. [file CNR2-7-e1930-s005.doc]

Supplementary Table 1: *In silico* prediction of the *MLH1* variants

| c.1592T>G | c.826A>T | c.423A>C | c.412C>A | c.407A>T | c.394G>A | c.2005G>A | c.1969A>T | c.1612T>G | c.889G>A | c.2002G>A | c.977T>C | c.655A>G | Variant |
| --- | --- | --- | --- | --- | --- | --- | --- | --- | --- | --- | --- | --- | --- |
| n/a | n/a | n/a | n/a | n/a | n/a | n/a | rs765363859 | rs1559575214 | rs63750736 | rs63750292 | rs63751049 | rs1799977 | RS |
| n/a | n/a | n/a | n/a | n/a | n/a | n/a | benign | benign | benign | benign | benign | benign | Polyphen |
| Missense | Missense | Synonymous | Missense | Missense | Missense | Missense | Missense | Missense | Missense | Missense | Missense | Missense | Molecular  consequence |
| n/a | n/a | n/a | n/a | n/a | n/a | n/a | Deleterious | Deleterious | Deleterious | Neutral | Deleterious | Neutral | PROVEAN PREDICTION (cutoff=-2.5) |
| n/a | n/a | n/a | n/a | n/a | n/a | n/a | Tolerated | Tolerated | Tolerated | Tolerated | Deleterious | Tolerated | SIFT Predction 0.05 CUT OFF |
| n/a | n/a | n/a | n/a | n/a | n/a | n/a | 22 likley benign | 24 likley benign | 23 likley benign | 23 likley benign | 24 likley benign | 17 likley benign | Cadd |
| n/a | n/a | n/a | n/a | n/a | n/a | n/a | 0.652 likley disease causing | 0.813 likley disease causing | 0.731 likely disease causing | 0.409 likely benign | 0.856 likley disease causing | 0.292 likely benign | Revel |
| n/a | n/a | n/a | n/a | n/a | n/a | n/a | 0.488 Tolerated | 0.845 damaging | 0.709 damaging | 0.692 damaging | 0.709 damaging | 0 tolerated | Meta LR |
| n/a | n/a | n/a | n/a | n/a | n/a | n/a | 0.578  medium | 0.707  medium | 0.772  medium | 0.576  medium | 0.91  medium | 0.255 low | Mutational assesor |
| n/a | n/a | n/a | n/a | n/a | n/a | n/a | n/a | uncertain | n/a | uncertain | benign | benign | Ensembl result |
| n/a | n/a | n/a | n/a | n/a | n/a | n/a | n/a | n/a | n/a | uncertain | benign | benign | ClinVar result |

Supplementary Table 2: *In silico* prediction of the *MSH2* variants

| c.2613G>A | c.2701G>A | c.2704G>A | c.2754G>A | c.2599G>T | c.2524G>A | c.1241T>G | c.842C>G | Variant |
| --- | --- | --- | --- | --- | --- | --- | --- | --- |
| n/a | n/a | n/a | n/a | n/a | Not Reported in ClinVar | rs587779078 | rs63749991 | RS |
| n/a | n/a | n/a | n/a | n/a | n/a | 0.756  possibly damaging | n/a | Polyphen |
| synonimous | missense | missense | synonimous | Non-sense | missense | missense | Non-sense | Molecular  consequence |
| n/a | n/a | n/a | n/a | n/a | Uncertain | Uncertain | pathogen | PROVEAN PREDICTION (cutoff=-2.5) |
| n/a | n/a | n/a | n/a | n/a | Neutral | 0 Deleterious | low freq | SIFT Predction 0.05 CUT OFF |
| n/a | n/a | n/a | n/a | n/a | Tolerated | 27 likely benign | Stop gaind | Cadd |
| n/a | n/a | n/a | n/a | n/a | n/a | 0.92 likely disease causing | n/a | Revel |
| n/a | n/a | n/a | n/a | n/a | n/a | 0.868 damaging | n/a | Meta LR |
| n/a | n/a | n/a | n/a | n/a | n/a | 0.865 medium | n/a | Mutational assesor |
| n/a | n/a | n/a | n/a | n/a | n/a | uncertain | n/a | Ensemble result |
| n/a | n/a | n/a | n/a | n/a | n/a | uncertain | pathogenic | ClinVar result |
